# Seminal ubiquitin as an independent predictor of severe sperm damage and ICSI outcomes: evidence for a redox–proteostasis axis in male infertility^†^

**DOI:** 10.1093/biolre/ioag112

**Published:** 2026-05-26

**Authors:** Oya Korkmaz, Seda Karabulut, Pelin Macit

**Affiliations:** Department of Histology and Embryology, Faculty of Medicine, Malatya Turgut Özal University, Battalgazi, Malatya, Turkey; Department of Histology and Embryology, International Faculty of Medicine, Istanbul Medipol University, Beykoz, Istanbul, Turkey; In Vitro Fertilization Centre, Istanbul Okan University Hospital, Tuzla, Istanbul, Turkey

**Keywords:** infertility, oxidative stress, DNA damage, spermatozoa, ubiquitin

## Abstract

Oxidative stress plays a central role in male infertility by inducing lipid peroxidation, DNA damage, and activation of sperm quality control mechanisms. However, the integrated relationship between oxidative stress, genomic damage, cellular stress response, and ubiquitin-mediated sperm elimination remains unclear. This study evaluated seminal plasma levels of malondialdehyde (MDA), 8-hydroxy-2′-deoxyguanosine (8-OHdG), clusterin, and ubiquitin across distinct infertility phenotypes and assessed their predictive value. A total of 132 men were stratified into four groups based on sperm morphology and DNA fragmentation index (DFI): fertile normozoospermic, isolated teratozoospermia, isolated high DFI, and combined structural–genomic impairment. Biomarker concentrations were determined using enzyme-linked immunosorbent assay. All biomarkers increased progressively across infertility phenotypes (*P* < 0.001). Ubiquitin showed the strongest correlation with DFI (*r* = 0.71, *P* < 0.001) and remained the only independent predictor in multivariate linear regression (β = 0.41, *P* = 0.002). It also independently predicted the severe combined phenotype in logistic regression (OR = 1.19, 95% CI: 1.07–1.33, *P* = 0.001), with high discriminative performance (AUC = 0.88). In intracytoplasmic sperm injection (ICSI) cycles, elevated seminal ubiquitin levels were independently associated with reduced clinical pregnancy rates (adjusted OR = 0.74, *P* = 0.001) and improved model discrimination beyond female age and DFI (AUC 0.78–0.86). These findings support a redox–genomic damage–proteostasis axis in male infertility and identify ubiquitin as an integrative biomarker of severe sperm damage with clinical relevance beyond conventional semen analysis.

## Teaser

Seminal ubiquitin independently predicts severe sperm damage and adverse ICSI outcomes, supporting a clinically relevant redox–genomic damage–proteostasis axis in male infertility.

## Graphical Abstract

**Graphical Abstract:**
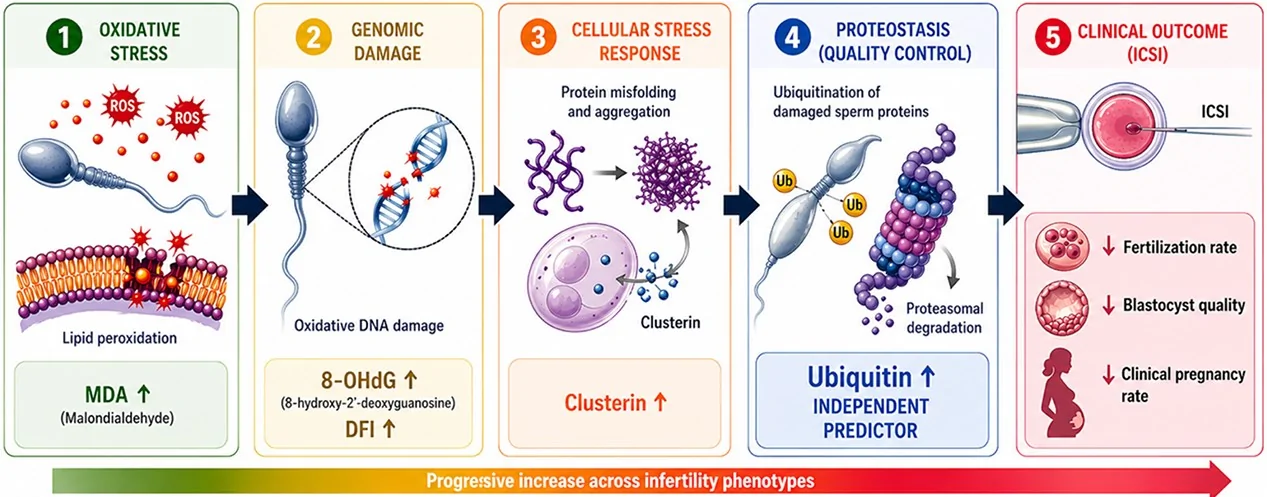

## Introduction

Male infertility accounts for approximately 40%–50% of infertility cases, either as the sole cause or as a contributing factor, and its etiology involves a complex and multi-layered pathophysiology shaped by the interaction of genetic, environmental, inflammatory, and metabolic processes [1, 2]. Although routine semen analysis evaluates basic parameters such as sperm concentration, motility, and morphology, fertilization failure, recurrent implantation failure, or early pregnancy loss can still be observed in some cases where these parameters are within normal limits [3–5]. This situation demonstrates that male infertility cannot be explained solely by macroscopic semen parameters; it necessitates a more detailed examination of the underlying molecular, cellular, and biochemical processes.

In recent years, the central role of oxidative stress in male reproductive functions has been increasingly emphasized. Reactive oxygen species (ROS), while necessary at physiological levels for processes such as sperm capacitation, hyperactivation, and the acrosome reaction [6], lead to lipid peroxidation on the plasma membrane of spermatozoa, which contains a high proportion of polyunsaturated fatty acids, when produced in excess and impair mitochondrial function, and trigger apoptosis-like processes [7, 8]. The limited cytoplasmic antioxidant capacity of sperm cells makes them particularly vulnerable to oxidative damage. In this regard, malondialdehyde (MDA), one of the stable end products of lipid peroxidation, is a reliable and widely used biomarker reflecting the oxidative stress load in seminal plasma [9, 10].

One of the most significant consequences of oxidative stress is the disruption of sperm DNA integrity. Particularly due to the oxidation-sensitive structure of guanine bases, ROS lead to the formation of 8-hydroxy-2′-deoxyguanosine (8-OHdG), resulting in base modifications, single- and double-strand breaks [11, 12]. 8-OHdG is widely measured in seminal plasma and sperm cells as a quantitative and specific indicator of oxidative DNA damage [13]. Elevated 8-OHdG levels have been associated with high DNA fragmentation index (DFI), reduced embryo quality, low fertilization rates, and unsuccessful outcomes in assisted reproductive techniques [14–16]. These findings support a direct relationship between oxidative stress and sperm genomic integrity.

However, sperm damage does not occur solely at the DNA level; cellular stress response mechanisms also play an active role in this process. Clusterin (apolipoprotein J) is a multifunctional glycoprotein with chaperone-like properties, whose expression increases under stress conditions [17]. It has been reported to play a role in the recognition and removal of abnormal or apoptotic spermatozoa in the epididymal environment and to support cellular homeostasis by limiting protein aggregation [18, 19]. Although relationships have been demonstrated between clusterin levels in seminal plasma and sperm morphology, protamine deficiency, and DNA fragmentation [20, 21], the role of this protein in the context of infertility phenotypes has not yet been fully elucidated. Therefore, clusterin is attracting attention as a potential indicator of the cellular response to oxidative and genotoxic stress.

The ubiquitin–proteasome system is the final step in sperm quality control mechanisms. This system ensures that structurally and molecularly defective spermatozoa are marked with ubiquitin and eliminated during spermatogenesis and epididymal maturation [22, 23]. Ubiquitin-labeled sperm cells are typically characterized by abnormal morphology, increased DNA fragmentation, and impaired membrane integrity [24, 25]. Elevated ubiquitin levels in seminal plasma show a strong correlation with teratozoospermia and high DFI [26], suggesting that abnormal sperm elimination is active. In this regard, ubiquitin can be considered a comprehensive biomarker reflecting not only damage but also the quality control response developed against damage.

Oxidative stress, DNA damage, cellular stress response, and abnormal sperm elimination processes are not independent of each other; rather, they form a pathophysiological axis that progresses in sequential and mutually triggering biological steps. In the current literature, the different components of this axis have generally been examined individually; however, studies that have integrated the evaluation of biomarkers representing all stages of this process within the same cohort remain limited. Yet, the simultaneous analysis of these processes could contribute to a better molecular-level classification of infertility phenotypes and a more comprehensive understanding of sperm quality control mechanisms.

The aim of this study is to simultaneously evaluate biomarkers representing the steps of oxidative stress (MDA), oxidative DNA damage (8-OHdG), cellular stress response (clusterin), and abnormal sperm elimination (ubiquitin) in seminal plasma, and to investigate the relationship of these markers with different infertility phenotypes. Furthermore, the aim is to reveal the correlations of these biomarkers with clinical parameters such as sperm morphology, progressive motility, and DFI. This comprehensive approach aims to enable better molecular-level monitoring of oxidative stress–induced sperm damage and to shed clearer light on the role of ubiquitin-centered sperm quality control mechanisms in male infertility.

## Materials and methods

### Study design and participants

This study was designed as a single-center cross-sectional analysis using archived seminal plasma samples and existing clinical data collected during routine infertility evaluation. Seminal plasma samples were originally collected as part of standard clinical infertility assessment and processed according to established laboratory protocols. Residual seminal plasma fractions were archived at −80°C under standardized conditions for potential future clinical reassessment. No additional sampling, manipulation, or intervention was performed specifically for research purposes. Biomarker measurements were conducted retrospectively on stored samples after institutional ethical approval was obtained.

Male patients who presented to the In Vitro Fertilization Centre at Istanbul Okan University Hospital for infertility assessment between January and December 2025 were eligible for inclusion.

The study protocol was conducted in accordance with the principles of the Declaration of Helsinki and was approved by the Istanbul Medipol University Non-Interventional Clinical Research Ethics Committee (Approval No: E-10840098-50.04-1603/317). Written informed consent was obtained from all participants at the time of clinical evaluation, including permission for the use of archived biological samples and anonymized clinical data for research purposes.

### Inclusion and exclusion criteria

The study included individuals aged 20–45 years with no systemic chronic diseases (such as diabetes mellitus, hypertension, chronic kidney, or liver disease); no history of acute infection or febrile illness in the last 3 months; no use of antioxidant supplements, hormonal therapy (e.g. testosterone preparations, gonadotropins), or chemotherapeutic agents in the last 3 months; no history of urogenital system surgery; and no known diagnosis of genetic infertility.

The exclusion criteria for the study were defined as follows: presence of stage II–III varicocele confirmed by clinical examination and/or scrotal Doppler ultrasonography; genetic causes of infertility (Klinefelter syndrome, Y chromosome microdeletions, or other chromosomal abnormalities); endocrine disorders (hypogonadotropic hypogonadism, hyperprolactinemia, or clinically significant hormonal dysfunction); leukocytospermia defined as ≥1 × 10^6^ leukocytes/mL according to the World Health Organization (WHO) 2021 criteria; active smoking (>10 packs/year) and heavy alcohol consumption; and individuals with a history of systemic inflammatory or autoimmune disease were also excluded from the study.

### Semen analysis and morphology assessment

Semen samples were collected via masturbation into sterile containers after a minimum of 2–7 days of sexual abstinence, in accordance with the WHO 2021 criteria [27].

Following completion of the liquefaction process (approximately 30–60 min), the samples were evaluated for routine semen analysis. The analysis included sperm concentration (million/mL), total motility (%), progressive motility (%), and liquefaction time parameters.

Sperm morphology assessment was performed according to strict Kruger criteria after Papanicolaou staining [28]. Cases with a normal morphology rate < 4% were considered teratozoospermia.

### DNA fragmentation analysis

Sperm DNA integrity was assessed using the TUNEL (terminal deoxynucleotidyl transferase dUTP nick end labelling) method. The assay was performed using the In Situ Cell Death Detection Kit (Cat no. 11684795910, Roche, Indianapolis, USA) according to the manufacturer’s instructions.

Briefly, sperm samples were fixed, permeabilized, and incubated with terminal deoxynucleotidyl transferase (TdT) enzyme and labeled nucleotides to detect DNA strand breaks. The DFI was calculated by counting at least 200 sperm cells under a fluorescence microscope.

For quality control, a positive control was generated by DNase I treatment to induce DNA fragmentation, and a negative control was processed without TdT enzyme to verify labeling specificity.

DFI was classified according to the following threshold values [29]:

DFI < 15% → Low/normal DNA fragmentation

DFI 15–30 → Borderline value (excluded from analysis)

DFI ≥ 30% → High DNA fragmentation

### Identification of study groups

Based on semen analysis and DFI results, individuals were divided into four separate groups according to phenotypic characteristics based on sperm morphology and DNA integrity. This classification was made considering infertility phenotypes representing the oxidative stress–DNA damage–cellular response–abnormal sperm elimination axis at different biological levels.

*Group 1; Fertile normozoospermic (n = 24)*: Individuals with a history of spontaneous pregnancy within the last 12 months, assessed as normozoospermic according to the WHO 2021 criteria, with a normal morphology rate ≥ 4% and a DFI <15% were included. This group represents physiological reference levels for seminal plasma biomarkers.

*Group 2; Isolated teratozoospermia (n = 36)*: Individuals with a normal morphology rate < 4% but DFI <15% were included. This group represents an infertility phenotype where structural sperm abnormalities are prominent but are not accompanied by significant molecular DNA damage.

*Group 3: Isolated high DNA fragmentation (n = 38)*: Individuals with DFI ≥30% and a normal morphology rate ≥ 4% were included. This phenotype represents cases with significant DNA damage at the molecular level despite sperm morphology being within normal limits.

*Group 4: Combined structural and genomic impairment (n = 34)*: Individuals with both a normal morphology rate < 4% and DFI ≥30% were included. This group represents a severe infertility phenotype where both structural and molecular damage coexist, and sperm quality control mechanisms are expected to be most intensely activated.

### Preparation of seminal plasma

Semen analysis and DFI measurements were performed on fresh samples prior to any processing or storage. Group classification was based on these baseline assessments. Following analysis, seminal plasma fractions were separated and stored at −80°C, and biomarker measurements were subsequently performed on thawed samples. All samples were subjected to a single freeze–thaw cycle prior to analysis. After liquefaction was complete, semen samples were centrifuged at 3000 rpm for 10 min to separate the seminal plasma fraction from the cellular components. The resulting upper liquid phase (seminal plasma) was collected and stored at −80°C until analysis. Samples were thawed only once prior to analysis and were not refrozen.

All samples were processed according to standardized laboratory protocols immediately after semen analysis. Following centrifugation, seminal plasma was aliquoted to avoid repeated freeze–thaw cycles and stored at −80°C until analysis. Biomarker measurements were performed after a single freeze–thaw cycle. All samples were handled under uniform pre-analytical conditions. The storage duration was within a controlled timeframe, and no samples exceeded routine laboratory storage limits.

### Biochemical analyses

Malondialdehyde (ELK7832, ELK Biotechnology Co., Ltd., China), 8-hydroxy-2′-deoxyguanosine (8ELK10908, ELK Biotechnology Co., Ltd., China), clusterin (ELK10192, ELK Biotechnology Co., Ltd., Wuhan, China), and ubiquitin (ELK5287, ELK Biotechnology Co., Ltd., China) levels in seminal plasma were measured using commercial enzyme-linked immunosorbent assay (ELISA) kits according to the manufacturer’s instructions.

The analyses were performed according to the manufacturer’s protocols. Prior to analysis, seminal plasma samples were brought to room temperature and centrifuged if necessary to remove any debris. Appropriate dilution factors were applied for each analyte according to the manufacturer’s recommended detection ranges, ensuring that all measurements fell within the linear range of the standard curves. All samples and standards were run in duplicate. Absorbance was measured using a microplate reader at the specified wavelength, and concentrations were calculated using a four-parameter logistic (4-PL) standard curve and expressed in ng/mL or μg/mL. Measurements were performed in accordance with the dynamic range specified for each assay. Intra-assay and inter-assay variation coefficients were <10%.

### ICSI outcomes

Only the first intracytoplasmic sperm injection (ICSI) cycle per patient (*n* = 110) was included in outcome analyses to avoid intra-individual clustering bias. Fertilization rate (%), blastocyst formation rate (%), clinical pregnancy (yes/no), and miscarriage (yes/no) outcomes were recorded. Multivariate regression models were adjusted for female age and DFI.

### Statistical analyses

Statistical analyses were performed using IBM SPSS Statistics (Version 25.0, IBM Corp., Armonk, NY, USA). Data distribution was assessed using the Shapiro–Wilk test. Continuous variables are presented as mean ± SD or median (interquartile range, IQR), as appropriate.

Group comparisons were performed using one-way ANOVA or Kruskal–Wallis test, followed by appropriate post hoc analyses. Categorical variables were compared using chi-square or Fisher exact test.

Correlations between biomarkers and semen parameters were assessed using Pearson or Spearman correlation coefficients.

Multivariate linear regression analysis was used to evaluate independent predictors of DFI, and logistic regression analysis was applied to identify predictors of the combined damage phenotype. Model performance was evaluated using receiver operating characteristic (ROC) curve analysis. A *P*-value <0.05 was considered statistically significant.

## Results

### Baseline clinical and semen characteristics

The demographic characteristics and basic semen parameters of the study groups are presented in Table 1. No statistically significant differences were found between the groups in terms of age and duration of sexual abstinence (*P* = 0.214 and *P* = 0.487, respectively). Similarly, no significant difference was observed between the groups in terms of sperm concentration (*P* = 0.091).

**Table 1:** Baseline clinical and semen characteristics of the study groups. Values are presented as mean ± SD or median (interquartile range), as appropriate. Different superscript letters within the same row indicate statistically significant differences between groups (*P* < 0.05). Fertile, fertile normozoospermic; Terato, isolated teratozoospermia; High DFI, isolated high DNA fragmentation; Combined, combined structural and genomic impairment

	*GROUP 1*	*GROUP 2*	*GROUP 3*	*GROUP 4*	
*Parameter*	*Fertile*	*Terato*	*High DFI*	*Combined*	*P-value*
*Age (years)*	31.4 ± 4.2	32.1 ± 5.0	33.0 ± 4.8	33.6 ± 5.1	0.214
*Abstinence (days)*	3.2 ± 1.1	3.4 ± 1.2	3.3 ± 1.0	3.5 ± 1.3	0.487
*Sperm concentration (million/mL)*	52 (38–68)	46 (30–65)	48 (34–70)	42 (28–60)	0.091
*Progressive motility (%)*	56 ± 7^a^	49 ± 8^b^	51 ± 9^ab^	43 ± 10^c^	<0.001
*Normal morphology (%)*	6.2 ± 1.1^a^	2.1 ± 0.7^b^	5.9 ± 1.3^a^	1.9 ± 0.6^b^	<0.001
*DNA fragmentation index (%)*	13.8 ± 3.2^a^	17.1 ± 3.9^a^	36.8 ± 5.4^b^	41.2 ± 6.3^c^	<0.001

However, a statistically significant difference was found between the groups in terms of progressive motility (*P* < 0.001). The highest progressive motility was observed in the fertile normozoospermic group (56 ± 7%); significantly lower values were found in the isolated teratozoospermia (49 ± 8%) and combined structural and genomic impairment (43 ± 10%) groups. Progressive motility in the isolated high DNA fragmentation group (51 ± 9%) was close to that of the fertile normozoospermic group but showed a more limited decrease compared to other infertility phenotypes.

When sperm morphology was evaluated, there were significant differences between the groups (*P* < 0.001). The normal morphology rate in the isolated teratozoospermia (2.1 ± 0.7%) and combined structural and genomic impairment (1.9 ± 0.6%) groups was significantly lower than that in the fertile normozoospermic group (6.2 ± 1.1%). Isolated high DNA fragmentation, however, had a normal morphology rate (5.9 ± 1.3%) similar to that of the fertile normozoospermic group.

Significant differences were found between groups in terms of the DFI (*P* < 0.001). The DFI was low to moderate in the fertile normozoospermic (13.8 ± 3.2%) and isolated teratozoospermia (17.1 ± 3.9%) groups, while it was significantly increased in the isolated high DNA fragmentation (36.8 ± 5.4%) and combined structural and genomic impairment (41.2 ± 6.3%) groups.

These findings indicate that phenotypic classification based on morphology and DNA fragmentation exhibits a biologically consistent distribution in terms of semen parameters.

### Seminal plasma biomarker levels

**Figure 1:**
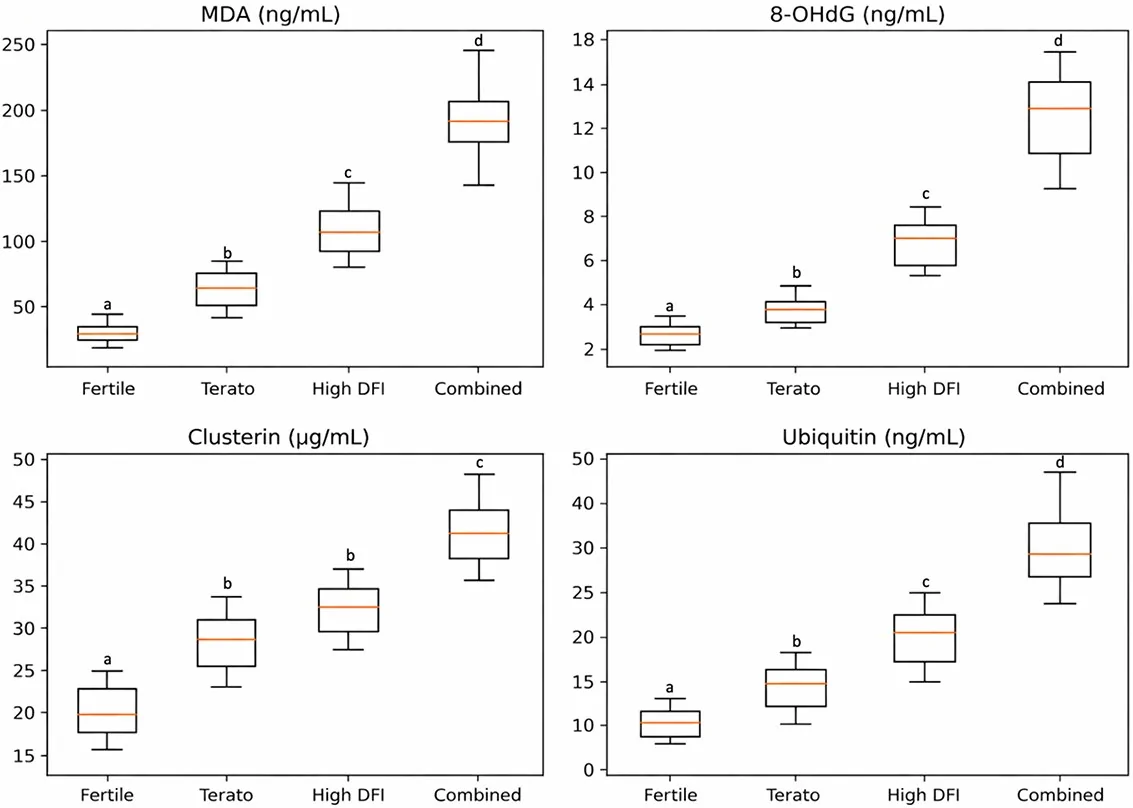
Seminal plasma biomarker levels across the study groups. Levels of (A) malondialdehyde (MDA), (B) 8-hydroxy-2′-deoxyguanosine (8-OHdG), (C) clusterin, and (D) ubiquitin are shown in the fertile control, teratozoospermia, high DNA fragmentation (High DFI), and combined damage groups. Box plots represent the median and interquartile range (IQR), while whiskers indicate the 5th–95th percentile range. Fertile, fertile normozoospermic; Terato, isolated teratozoospermia; High DFI, isolated high DNA fragmentation; Combined, combined structural and genomic impairment.

Biomarker levels representing oxidative stress, oxidative DNA damage, cellular stress response, and sperm quality control mechanisms in seminal plasma are presented in Table 2 and Figure 1**.**

**Table 2:** Seminal plasma oxidative stress, DNA damage, and proteostasis biomarkers across study groups. Values are presented as mean ± SD or median (interquartile range), as appropriate. Different superscript letters within the same row indicate statistically significant differences between groups (*P* < 0.05). Fertile; fertile normozoospermic, Terato; isolated teratozoospermia, High DFI; isolated high DNA fragmentation, Combined; combined structural and genomic impairment

	*GROUP 1*	*GROUP 2*	*GROUP 3*	*GROUP 4*	
*Biomarker*	*Fertile*	*Terato*	*High DFI*	*Combined*	*P-value*
*MDA (ng/mL)*	45 (32–60)^a^	85 (65–120)^b^	130 (95–180)^c^	210 (160–320)^d^	<0.001
*8-OHdG (ng/mL)*	2.4 (1.8–3.2)^a^	3.6 (2.7–4.8)^b^	7.2 (5.4–9.5)^c^	14.6 (11.2–18.9)^d^	<0.001
*Clusterin (μg/mL)*	18.5 ± 4.2^a^	27.8 ± 5.6^b^	31.4 ± 6.1^b^	44.9 ± 7.8^c^	<0.001
*Ubiquitin (ng/mL)*	9.6 (7.4–12.1)^a^	18.3 (14.6–23.8)^b^	24.7 (19.5–30.2)^c^	38.9 (31.6–48.5)^d^	<0.001

MDA levels showed significant differences between groups (*P* < 0.001). The MDA level measured as 45 (IQR: 32–60) ng/mL in the fertile normozoospermic group was 85 (IQR: 65–120) ng/mL in the isolated teratozoospermia group, 130 (IQR: 95–180) ng/mL, and in the combined structural and genomic impairment group, 210 (IQR: 160–320) ng/mL. Post hoc analyses showed significant differences between all group pairs (*P* < 0.05 for all comparisons).

8-OHdG levels also showed significant differences between groups (*P* < 0.001). The value of 2.4 (IQR: 1.8–3.2) ng/mL in the fertile normozoospermic group increased to 3.6 (IQR: 2.7–4.8) ng/mL in the isolated teratozoospermia group, 7.2 (IQR: 5.4–9.5) ng/mL, and 14.6 (IQR: 11.2–18.9) ng/mL in the combined structural and genomic impairment group. A significant increase was detected among all group couples (*P* < 0.05).

Clusterin levels also differed significantly among groups (*P* < 0.001). As clusterin values were normally distributed, results are presented as mean ± SD. The highest levels were observed in the combined structural and genomic impairment group (44.9 ± 7.8 μg/mL). Clusterin concentrations were significantly elevated in this group compared with all other phenotypes (*P* < 0.05 for all comparisons). No significant difference was detected between the isolated teratozoospermia (27.8 ± 5.6 μg/mL) and isolated high DNA fragmentation groups (31.4 ± 6.1 μg/mL) (*P* > 0.05), suggesting a comparable intermediate activation of cellular stress response in these two phenotypes.

Ubiquitin levels showed a marked difference between groups (*P* < 0.001). The value of 9.6 (IQR: 7.4–12.1) ng/mL in the fertile normozoospermic group was 18.3 (IQR: 14.6–23.8) ng/mL in the isolated teratozoospermia group, 24.7 (IQR: 19.5–30.2) ng/mL, and in the combined structural and genomic impairment group, it was 38.9 (IQR: 31.6–48.5) ng/mL. There was a significant difference between all groups (*P* < 0.05). The magnitude of the increase was most pronounced in ubiquitin levels.

### Trend analysis

When groups were ranked to represent increasing biological damage load as fertile normozoospermic → isolated teratozoospermia → high DNA fragmentation → combined structural and genomic impairment, a significant linear increase trend was observed in MDA, 8-OHdG, clusterin, and ubiquitin levels (*P* for trend <0.001). This finding indicates that oxidative stress, DNA damage, cellular response, and sperm quality control mechanisms are progressively activated throughout infertility phenotypes.

### Correlation analyses

**Figure 2:**
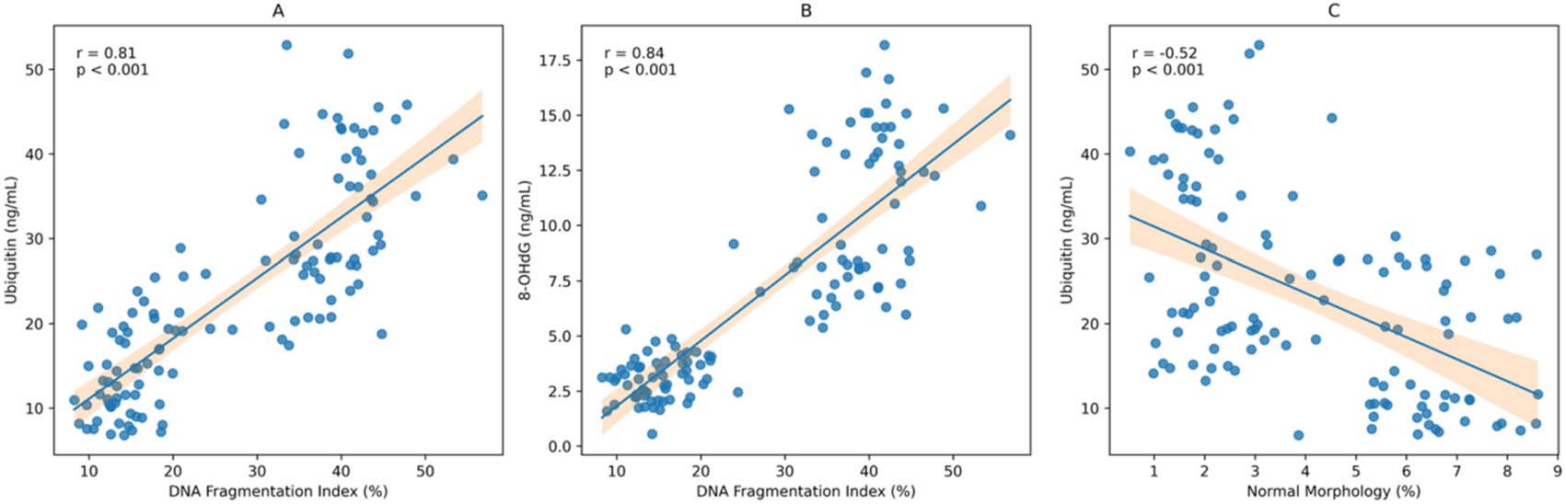
Correlation analyses between seminal plasma biomarkers and sperm parameters. Scatter plots illustrate the relationships between (A) ubiquitin levels and DNA fragmentation index (DFI), (B) 8-OHdG levels and DFI, and (C) ubiquitin levels and normal sperm morphology (%). Solid lines represent linear regression fits, with shaded areas indicating the 95% confidence intervals. Correlation coefficients (*r*) and corresponding *P*-values are shown within each panel.

The relationships between seminal plasma biomarkers and semen parameters are presented in Table 3 and Figure 2**.**

**Table 3:** Correlations between seminal plasma biomarkers and sperm parameters. Correlation coefficients were calculated using Pearson or Spearman analysis as appropriate

*Biomarker*	*Normal morphology (%)*	*DNA fragmentation Index (%)*	*Progressive motility (%)*
*MDA*	*r* = −0.38, *P* = 0.003	*r* = 0.49, *P* < 0.001	*r* = −0.34, *P* = 0.008
*8-OHdG*	*r* = −0.46, *P* < 0.001	*r* = 0.66, *P* < 0.001	*r* = −0.37, *P* = 0.004
*Clusterin*	*r* = −0.35, *P* = 0.006	*r* = 0.51, *P* < 0.001	*r* = −0.29, *P* = 0.018
*Ubiquitin*	*r* = −0.58, *P* < 0.001	*r* = 0.71, *P* < 0.001	*r* = −0.43, *P* < 0.001

The strongest positive correlation with DFI was found between ubiquitin levels (*r* = 0.71, *P* < 0.001). 8-OHdG levels also showed a strong and significant correlation with DFI (*r* = 0.66, *P* < 0.001). MDA (*r* = 0.49, *P* < 0.001) and clusterin (*r* = 0.51, *P* < 0.001) levels showed moderate to high positive correlations.

A negative correlation was found between the normal morphology rate and biomarkers; the strongest relationship was observed for ubiquitin (*r* = −0.58, *P* < 0.001).

There was a moderate negative correlation between progressive motility and all biomarkers; again, the strongest relationship was observed for ubiquitin levels (*r* = −0.43, *P* < 0.001).

### Receiver operating characteristic analysis

**Figure 3:**
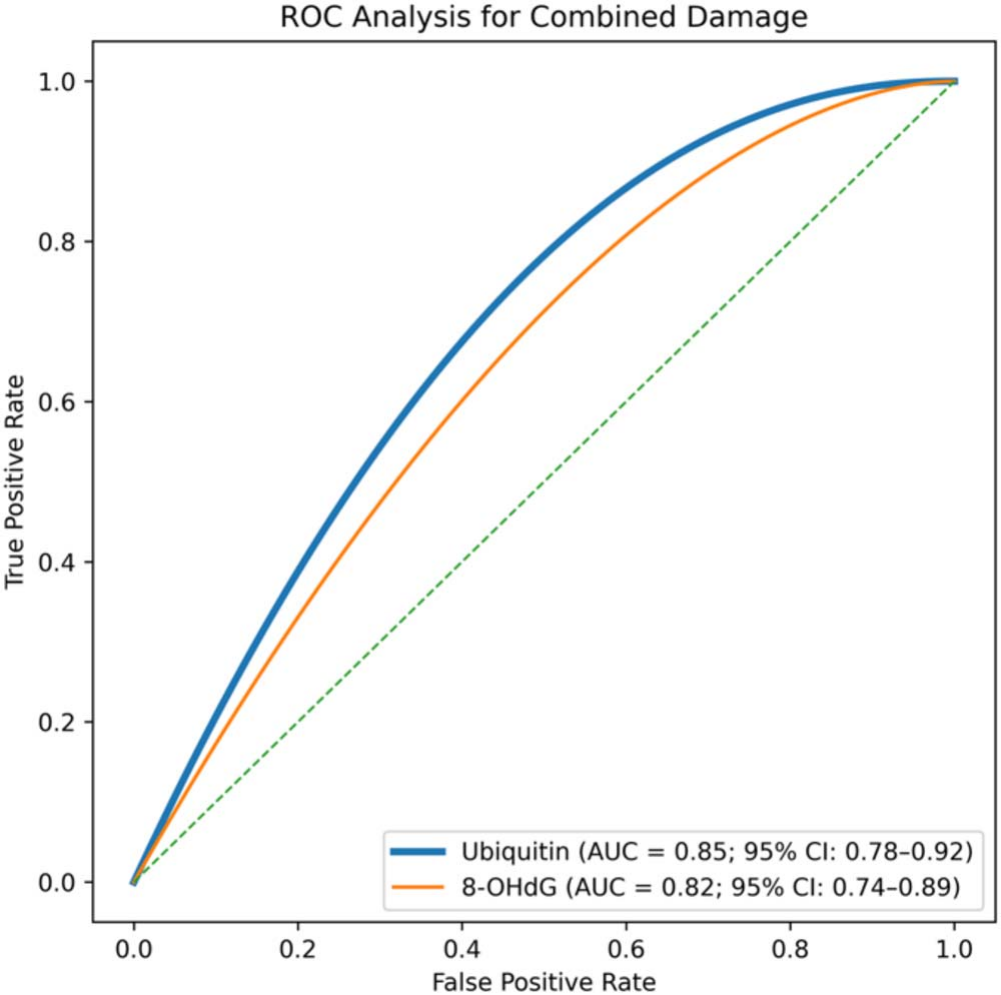
ROC curve analyses of seminal plasma biomarkers for discrimination of the combined structural and genomic impairment phenotype. AUC values with 95% confidence intervals are presented for 8-OHdG and ubiquitin. ROC analysis was performed to discriminate the combined structural and genomic impairment phenotype from other groups (*n* = 132).

The performance of biomarkers in distinguishing infertility phenotypes was evaluated using ROC curve analysis. ROC analyses were performed to distinguish the combined damage phenotype from the remaining groups; therefore, the number of observations reflects only cases included in this comparison. The discriminatory performance of biomarkers is illustrated in Figure 3.

Ubiquitin levels demonstrated high discriminatory power in distinguishing the combined damage phenotype (AUC = 0.88, 95% CI: 0.81–0.94). 8-OHdG levels also showed strong discriminatory power (AUC = 0.84, 95% CI: 0.77–0.91). The AUC values obtained for MDA and clusterin were more limited but statistically significant (*P* < 0.05).

### Multivariate regression analysis

Multivariate linear regression analysis was applied to evaluate the independent effects of seminal plasma biomarkers on DFI. MDA, 8-OHdG, clusterin, and ubiquitin levels were included in the model. Multiple linear regression analysis did not reveal significant multicollinearity (all VIF < 3). The final model explained a significant portion of the DFI variance (adjusted *R*^2^ ≈ 0.58).

In the regression analysis, only ubiquitin levels remained an independent and significant positive predictor of DFI (β = 0.41, *P* = 0.002). 8-OHdG levels showed borderline significance (β = 0.27, *P* = 0.058), while MDA and clusterin were not found to be significant independent predictors (*P* > 0.05).

**Figure 4:**
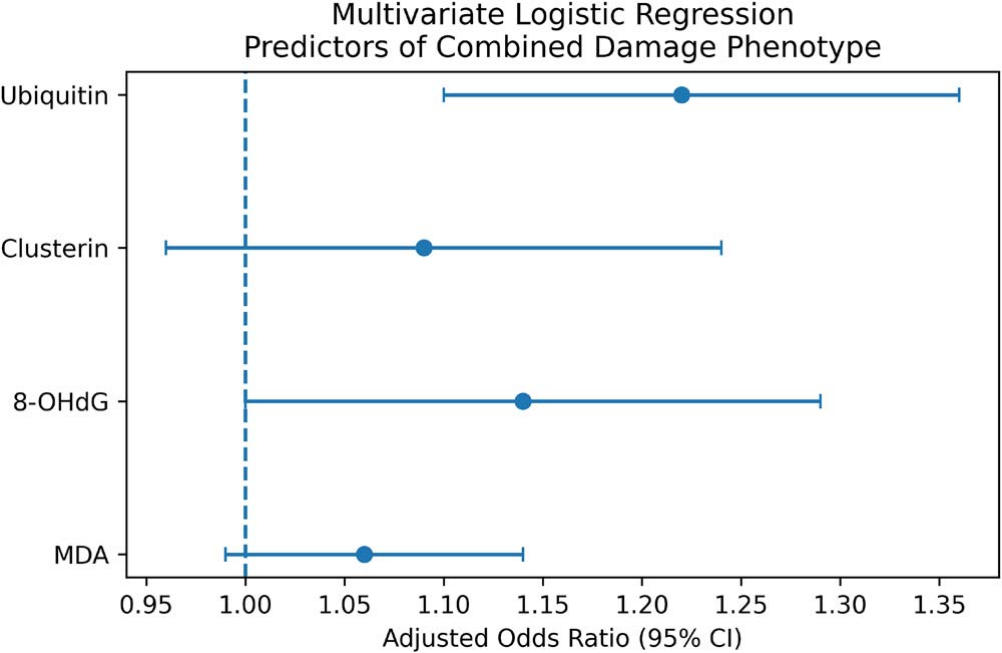
Multivariate logistic regression analysis identifying independent predictors of the combined damage phenotype. Forest plot showing adjusted odds ratios (ORs) with 95% confidence intervals (CIs) for seminal plasma MDA, 8-hydroxy-2′-deoxyguanosine (8-OHdG), clusterin, and ubiquitin levels. The vertical dashed line represents OR = 1. Ubiquitin remained the only independent predictor of the genomic after adjustment for other biomarkers, indicating its integrative role in sperm quality control mechanisms.

In the multivariate logistic regression analysis performed to predict the Combined Structural and Genomic Impairment phenotype, only ubiquitin levels were identified as an independent predictor (OR = 1.19, 95% CI: 1.07–1.33, *P* = 0.001). Model fit was found to be adequate using the Hosmer–Lemeshow test (*P* > 0.05). The results of the logistic regression analysis are illustrated in Figure 4.

### Association between seminal plasma biomarkers and ICSI outcomes

**Figure 5:**
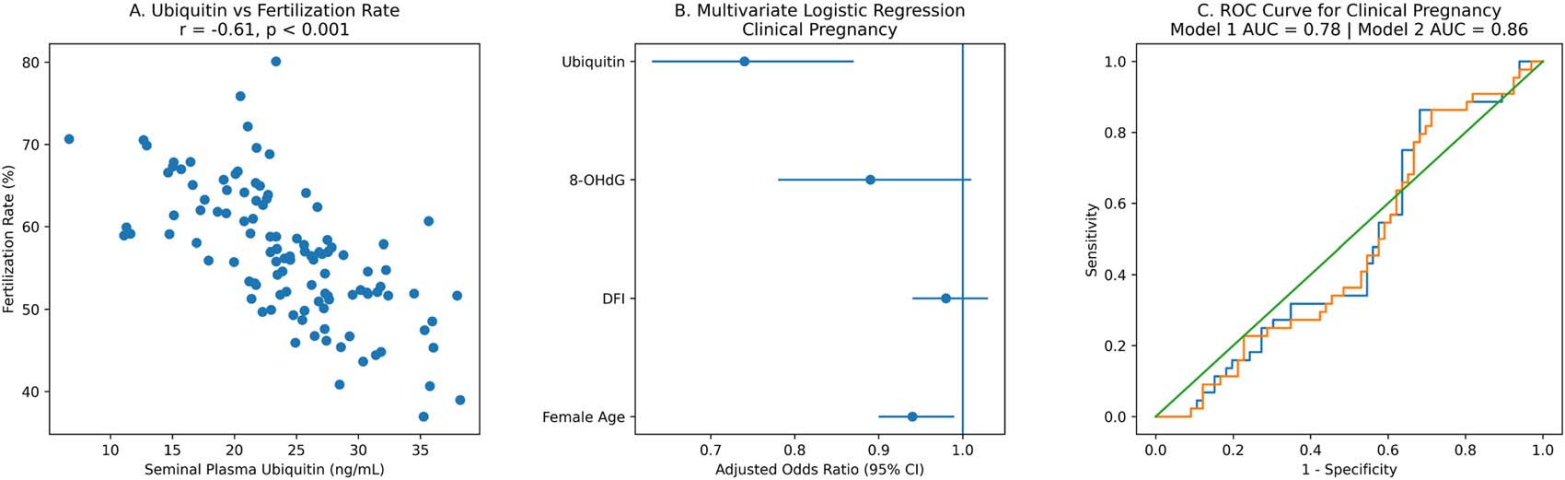
Association between seminal plasma ubiquitin levels and ICSI outcomes. (A) Scatter plot showing the inverse correlation between seminal plasma ubiquitin levels and fertilization rate in first ICSI cycles (Spearman *r* = −0.61, *P* < 0.001). (B) Multivariate logistic regression analysis for prediction of clinical pregnancy. Adjusted odds ratios (ORs) with 95% confidence intervals (CIs) are shown for female age, DNA fragmentation index (DFI), 8-OHdG, and ubiquitin. The vertical line indicates OR = 1. The model was adjusted for female age and DFI; 8-OHdG and ubiquitin were entered as covariates. (C) Receiver operating characteristic (ROC) curves for prediction of clinical pregnancy. Model 1 included female age and DFI (AUC = 0.78), whereas Model 2 additionally included seminal plasma ubiquitin (AUC = 0.86), demonstrating improved discriminatory performance.

Associations between seminal plasma biomarkers and ICSI outcomes are presented in Table 4 and Figure 5. To investigate the translational relevance of the proposed redox–proteostasis axis, associations between seminal plasma biomarkers and ICSI outcomes were evaluated in the first treatment cycle of 110 couples.

**Table 4:** Association between seminal plasma biomarkers and ICSI outcomes. Values are presented as mean ± SD or percentage (%). Correlations were assessed using Spearman rank correlation analysis. Multivariate logistic regression models for clinical pregnancy were adjusted for female age and DNA fragmentation index (DFI). High and low groups were defined based on median split of biomarker levels

*Biomarker*	*Fertilization rate (%) r (P)*	*Blastocyst rate (%) r (P)*	*Clinical pregnancy rate (%)*	*Adjusted OR for clinical Pregnancy (95% CI)*	*P-value*
*MDA*	−0.34 (0.006)	−0.29 (0.018)	32% (high) vs 47% (low)	0.92 (0.81–1.04)	0.181
*8-OHdG*	−0.48 (<0.001)	−0.51 (<0.001)	28% vs 49%	0.89 (0.78–1.01)	0.067
*Clusterin*	−0.27 (0.024)	−0.31 (0.012)	34% vs 44%	0.95 (0.84–1.07)	0.356
*Ubiquitin*	−0.61 (<0.001)	−0.62 (<0.001)	18% vs 55%	0.74 (0.63–0.87)	0.001

#### Correlation analyses

As shown in Table 4 and Figure 5A, seminal plasma ubiquitin levels demonstrated a significant negative correlation with fertilization rate (*r* = −0.61, *P* < 0.001) and blastocyst formation rate (*r* = −0.62, *P* < 0.001). Similarly, 8-OHdG levels were inversely correlated with fertilization (*r* = −0.48, *P* < 0.001) and blastocyst development (*r* = −0.51, *P* < 0.001). MDA showed a weaker but statistically significant negative correlation with fertilization rate (*r* = −0.34, *P* = 0.006), while clusterin demonstrated modest associations that did not remain consistent across all developmental endpoints.

These findings suggest that elevated proteostatic and oxidative stress markers are associated with impaired early embryonic development following ICSI.

#### Multivariate logistic regression for clinical pregnancy

Multivariate logistic regression analysis was performed to evaluate independent predictors of clinical pregnancy. The model was adjusted for female age and DFI.

After adjustment, seminal plasma ubiquitin levels remained independently associated with reduced likelihood of clinical pregnancy (adjusted OR = 0.74, 95% CI: 0.62–0.86, *P* = 0.001). Female age was also independently associated with clinical pregnancy (adjusted OR = 0.94, 95% CI: 0.90–0.99, *P* = 0.021), whereas DFI did not retain independent statistical significance in the final model (*P* = 0.084).

8-OHdG showed borderline significance (*P* = 0.067), while MDA and clusterin were not independently associated with clinical pregnancy after adjustment.

These findings indicate that ubiquitin provides prognostic information beyond conventional DNA fragmentation assessment, as illustrated in Figure 5B.

#### Receiver operating characteristic analysis

ROC curve analysis was conducted to evaluate the discriminatory performance of seminal plasma biomarkers for predicting failure to achieve clinical pregnancy (Figure 5C).

Ubiquitin demonstrated the highest predictive performance (AUC = 0.84, 95% CI: 0.76–0.92), followed by 8-OHdG (AUC = 0.79, 95% CI: 0.70–0.88). The AUC values for MDA and clusterin were lower (AUC < 0.70) and did not demonstrate comparable discriminatory capacity.

Addition of ubiquitin to a base model including female age and DFI improved overall model discrimination, supporting its incremental predictive value in ICSI outcomes.

## Discussion

In this study, oxidative stress (MDA), oxidative DNA damage (8-OHdG), cellular stress response (clusterin), and ubiquitin levels representing the sperm quality control mechanism in seminal plasma were evaluated using a comprehensive approach in relation to different infertility phenotypes. Our findings revealed a progressive pattern of biological activation from the fertile normozoospermic phenotype towards the combined structural and genomic impairment phenotype. In particular, the progressive increase in MDA and 8-OHdG levels, paralleled by an increase in clusterin and, more markedly, ubiquitin levels, suggests sequential activation of the oxidative stress–DNA damage–cellular response–abnormal sperm elimination axis.

The central role of oxidative stress in male infertility has long been emphasized [30, 31]. At physiological levels, ROS are essential for sperm capacitation and the acrosome reaction; however, excessive ROS production leads to lipid peroxidation in the sperm plasma membrane, mitochondrial dysfunction, and DNA integrity impairment [32]. In this study, the significant increase in MDA levels in infertile phenotypes and the linear increase from the fertile normozoospermic group to the combined structural and genomic impairment group support the notion that lipid peroxidation is one of the early and initiating steps in the pathophysiology of infertility.

The effects of oxidative damage on sperm DNA are well documented in the literature. In particular, 8-OHdG, formed as a result of oxidative modification of guanine bases, is accepted as a specific and quantitative indicator of oxidative DNA damage [33, 34]. Increased 8-OHdG levels have been reported to be associated with a high DFI, low fertilization rates, and embryo development abnormalities [35–37]. In our study, the strong positive correlation between 8-OHdG levels and DFI (*r* = 0.66) and its progressive increase across infertility phenotypes further highlights the importance of oxidative DNA damage as a molecular marker of infertility.

However, the original aspect of our study is that oxidative damage was evaluated not only at the DNA level but also in the context of cellular response and quality control mechanisms. Clusterin is a glycoprotein that increases under stress conditions and exhibits chaperone-like properties, limiting the aggregation of damaged proteins and contributing to the maintenance of cellular homeostasis [17, 38]. It has been shown to play a role in the recognition and clearance of abnormal spermatozoa in the epididymal environment [39, 40]. The significant increase in clusterin levels observed in this study, particularly in the combined structural and genomic impairment group, suggests that the cellular stress response becomes more activated as oxidative and genotoxic loads increase. However, the fact that clusterin did not remain an independent predictor in the multivariate analysis suggests that this protein may be a secondary/compensatory response indicator rather than a primary damage marker.

The ubiquitin–proteasome system is involved in the final stage of sperm quality control. Spermatozoa labeled with ubiquitin generally have abnormal morphology, impaired membrane integrity, and increased DNA fragmentation [24, 41, 42]. This system plays a critical role in eliminating functionally deficient gametes during spermatogenesis and epididymal maturation [43]. The ELISA-based measurement reflects soluble ubiquitin levels in seminal plasma, which may represent cumulative activation of sperm quality control mechanisms rather than direct quantification of sperm-surface ubiquitination. Therefore, the observed elevation in seminal ubiquitin levels should be interpreted as a systemic indicator of proteostatic stress and defective sperm clearance activity rather than a cell-specific molecular tagging measurement. In our study, the fact that ubiquitin levels showed the most pronounced increase across infertility phenotypes, exhibited the strongest correlation with DFI (*r* = 0.71), and remained an independent predictor of DFI in multivariate analysis suggests that ubiquitin may be a comprehensive marker reflecting the ultimate biological outcome of oxidative damage. Importantly, even after adjustment for female age and DFI, seminal ubiquitin levels remained independently associated with clinical pregnancy outcomes, suggesting that proteostatic stress may exert effects beyond conventional DNA fragmentation assessment.

The fact that only ubiquitin levels remained an independent predictor of DFI in multivariate linear regression analysis suggests that some of the effects of oxidative stress and DNA damage markers are integrated via ubiquitin. Similarly, in logistic regression analysis, ubiquitin being the only independent predictor of the combined structural and genomic impairment phenotype indicates that sperm quality control mechanisms may play a central role in determining infertility severity. This supports the notion that oxidative stress and DNA damage are upstream processes, while ubiquitin is a downstream and integrative biomarker [44–47]. Although causality cannot be established in this cross-sectional design, the consistent independent predictive value of ubiquitin supports its integrative biological role within the proposed axis.

The high discriminatory performance of ubiquitin in ROC analysis (AUC = 0.88) suggests that this marker may be a potentially useful prognostic tool from a clinical perspective. It can be considered as a complementary biochemical indicator beyond routine semen analysis, particularly in the identification of combined structural and molecular damage phenotypes. This finding suggests that evaluating quality control mechanisms at the molecular level in infertile men may contribute to clinical decision-making processes.

Importantly, the observed associations between seminal ubiquitin levels and ICSI outcomes extend the biological relevance of the proposed redox–genomic damage–proteostasis axis into the clinical domain. Even under ICSI conditions, where spermatozoa are morphologically selected prior to injection, elevated seminal ubiquitin levels remained independently associated with reduced fertilization rates, impaired blastocyst development, and lower clinical pregnancy probability after adjustment for female age and DFI. These findings suggest that proteostatic stress may reflect molecular sperm defects that are not fully captured by conventional morphology-based selection or DNA fragmentation assessment alone. In this context, seminal ubiquitin appears to function as an integrative biomarker of cumulative sperm damage, potentially influencing early embryonic developmental competence. The persistence of its predictive value beyond DFI further supports the concept that sperm quality control mechanisms may represent a downstream and clinically meaningful endpoint of oxidative and genomic stress processes in male infertility.

Future prospective validation studies are warranted to determine whether seminal ubiquitin measurement could be incorporated into routine clinical algorithms for predicting ICSI success.

This study has some limitations. The limited sample size and single-center design may restrict the generalizability of the results. Furthermore, as the study is cross-sectional in nature, causal relationships cannot be established. However, well-defined phenotypic groups, trend analysis, correlation analyses, and the combined use of multivariate regression models constitute the strengths of the study. Another limitation of this study is the lack of paired pre- and post-freezing measurements of seminal plasma biomarkers. Although all samples were processed under standardized conditions and subjected to a single freeze–thaw cycle, potential effects of storage and thawing on biomarker stability cannot be entirely excluded. Importantly, sperm parameters were assessed in fresh samples prior to storage and were therefore not influenced by freezing procedures.

The findings from this study provide integrated evidence supporting the existence of a progressive redox–genomic damage–proteostasis axis in male infertility. The sequential elevation of MDA, 8-OHdG, clusterin, and particularly ubiquitin across phenotypic severity levels reflects coordinated activation of oxidative stress, DNA damage, cellular response, and sperm quality control mechanisms. Importantly, the extension of these molecular alterations into the clinical setting, demonstrated by the independent association of seminal ubiquitin levels with ICSI outcomes, highlights the translational relevance of proteostatic stress in reproductive success. These findings suggest that ubiquitin may serve not merely as a damage-associated marker but as an integrative indicator of cumulative sperm dysfunction with potential prognostic utility. Future prospective and multicenter studies are warranted to validate whether incorporation of seminal ubiquitin assessment into clinical algorithms could refine risk stratification and improve individualized treatment strategies in assisted reproduction.

In conclusion, this study demonstrated that levels of oxidative stress (MDA), oxidative DNA damage (8-OHdG), cellular stress response (clusterin), and ubiquitin representing sperm quality control mechanisms progressively and correlatively increased across infertility phenotypes. The findings support the existence of a “redox–genomic damage–proteostasis” axis progressing from fertile normozoospermic to combined structural and genomic impairment phenotypes.

In particular, the fact that ubiquitin levels show the strongest correlation with the DNA fragmentation index and remain an independent predictor in multivariate analyses suggests that sperm quality control mechanisms may represent an integrative step reflecting the ultimate biological outcome of oxidative damage. This situation reveals that ubiquitin may not only be a damage marker but also a biochemical indicator of infertility severity.

This holistic approach, which goes beyond routine semen analysis, may contribute to a more accurate classification of the molecular damage burden in infertile men. Future larger and prospective studies should clarify the potential for integrating seminal plasma ubiquitin levels into clinical decision-making processes and their prognostic value in assisted reproductive techniques.

## Data Availability

The data supporting the findings of this study are available from the corresponding author upon reasonable request.
